# Beyond the Hype—The Actual Role and Risks of AI in Today’s Medical Practice: Comparative-Approach Study

**DOI:** 10.2196/49082

**Published:** 2024-01-22

**Authors:** Steffan Hansen, Carl Joakim Brandt, Jens Søndergaard

**Affiliations:** 1 Research Unit of General Practice Institution of Public Health University of Southern Denmark Odense Denmark

**Keywords:** AI, artificial intelligence, ChatGPT-4, Microsoft Bing, general practice, ChatGPT, chatbot, chatbots, writing, academic, academia, Bing

## Abstract

**Background:**

The evolution of artificial intelligence (AI) has significantly impacted various sectors, with health care witnessing some of its most groundbreaking contributions. Contemporary models, such as ChatGPT-4 and Microsoft Bing, have showcased capabilities beyond just generating text, aiding in complex tasks like literature searches and refining web-based queries.

**Objective:**

This study explores a compelling query: can AI author an academic paper independently? Our assessment focuses on four core dimensions: relevance (to ensure that AI’s response directly addresses the prompt), accuracy (to ascertain that AI’s information is both factually correct and current), clarity (to examine AI’s ability to present coherent and logical ideas), and tone and style (to evaluate whether AI can align with the formality expected in academic writings). Additionally, we will consider the ethical implications and practicality of integrating AI into academic writing.

**Methods:**

To assess the capabilities of ChatGPT-4 and Microsoft Bing in the context of academic paper assistance in general practice, we used a systematic approach. ChatGPT-4, an advanced AI language model by Open AI, excels in generating human-like text and adapting responses based on user interactions, though it has a knowledge cut-off in September 2021. Microsoft Bing's AI chatbot facilitates user navigation on the Bing search engine, offering tailored search

**Results:**

In terms of relevance, ChatGPT-4 delved deeply into AI’s health care role, citing academic sources and discussing diverse applications and concerns, while Microsoft Bing provided a concise, less detailed overview. In terms of accuracy, ChatGPT-4 correctly cited 72% (23/32) of its peer-reviewed articles but included some nonexistent references. Microsoft Bing’s accuracy stood at 46% (6/13), supplemented by relevant non–peer-reviewed articles. In terms of clarity, both models conveyed clear, coherent text. ChatGPT-4 was particularly adept at detailing technical concepts, while Microsoft Bing was more general. In terms of tone, both models maintained an academic tone, but ChatGPT-4 exhibited superior depth and breadth in content delivery.

**Conclusions:**

Comparing ChatGPT-4 and Microsoft Bing for academic assistance revealed strengths and limitations. ChatGPT-4 excels in depth and relevance but falters in citation accuracy. Microsoft Bing is concise but lacks robust detail. Though both models have potential, neither can independently handle comprehensive academic tasks. As AI evolves, combining ChatGPT-4’s depth with Microsoft Bing’s up-to-date referencing could optimize academic support. Researchers should critically assess AI outputs to maintain academic credibility.

## Introduction

Artificial intelligence’s (AI) journey has been nothing short of incredible. Starting with its early days of rule-based systems, we have seen it grow and mature, stepping into the realm of machine learning, and more recently, diving into deep learning. This transformative journey has shaken up a lot of sectors, but health care is where AI has truly left an indelible mark.

Today, algorithms can spot issues in our x-rays or magnetic resonance imaging, sometimes even better than our seasoned doctors [1]. AI does not just stop there; it even gives us a heads-up on potential life-threatening situations in intensive care units, predicting conditions like septic shock hours before they occur. The world of drug discovery is moving faster than ever, thanks to AI’s helping hand [2]. However, as with most things, there are issues. There are big questions about how we protect our data and ensure different health record systems talk to each other [3], not to mention the lingering worries about biases in AI and the sometimes uneasy feeling of trusting a machine we do not fully “get” [4].

When you look at the big picture, we see ground-breaking models like GPT-3, ChatGPT-4 [5,6], and Microsoft Bing [7] making waves. They are not just about churning out text. They are doing things we had never imagined, like assisting in literature searches or refining our everyday web-based searches [8]. Their accomplishments in challenges, such as the Turing Test [9] and the LAMBADA (LAnguage Modeling Broadened to Account for Discourse Aspects) tasks [10], just go on to show how capable they are. Comparing powerhouses like ChatGPT-4 and Bing is not just for fun; it gives us a glimpse into where AI’s language abilities might be headed, and with new kids on the block like Google Bard, the sky is the limit [11]. Writing an academic paper, though? That is still a world where the human touch shines. From combing through mountains of literature to connecting the dots in innovative ways, it is a craft that demands the very best of us, but here is a thought: given how far AI has come, could it, one day, pen down an academic masterpiece on its own? This paper is all about that tantalizing question.

As we embark on this exploration, we will keenly assess a few critical dimensions:

- Relevance: can AI ensure that its response precisely addresses the prompt and brings to the table information that is truly pertinent to the question or topic?
- Accuracy: how reliable is AI in delivering information that is not just factually correct but also up-to-date with the current pulse of the academic field?
- Clarity: when we read what is written by AI, does it resonate with clarity, coherence, and a logical flow of ideas, all presented with precise and unambiguous language?
- Tone and style: given the seriousness of academic papers, can AI match the appropriate tone and style, ensuring it resonates with the formality and professionalism we expect to see in academic texts?

We are diving deep to see if AI can muster up the relevance, accuracy, clarity, and tone we associate with academic work, and of course, while we probe these questions, we are not losing sight of the overarching ethics and practicality of inviting AI into the revered domain of academic writing.

## Methods

### Ethical Considerations

In Denmark, ethical committee approval is only mandatory for studies that include trials involving liveborn human individuals, human gametes intended for fertilization, fertilized human eggs, embryonic cells and embryos, tissue, cells and genetic material from humans, embryos, etc, or deceased persons. Also included are clinical trials of medicines in humans and clinical trials of medical devices. Hence, our study did not require approval from an ethical committee.

### Overview

In this methods section, we have detailed the approach taken to evaluate and compare the performance of ChatGPT-4 and Microsoft Bing in the context of assisting with an academic paper in the realm of general practice. This section outlines the data collection process, prompt design, evaluation criteria, and analysis of the AI-generated responses.

### Models

#### ChatGPT-4

ChatGPT-4 is an advanced AI language model developed by OpenAI [5], based on the ChatGPT-4 architecture. It is designed to generate human-like text and engage in interactive conversations with users. Trained on a vast data set, ChatGPT-4 demonstrates a strong understanding of context, language, and reasoning abilities. When using GPT-4, it is important to highlight that during a conversation, the information and discussion are dynamically shaped throughout the interaction. Indeed, GPT-4 can respond by incorporating the information the user provides, potentially leading to different outcomes even for users with similar queries. This dynamic nature is crucial for understanding how a large language model like GPT-4 operates.

Although ChatGPT-4 can perform various tasks, such as answering questions, providing recommendations, and generating content, it has a knowledge cut-off date of September 2021. This means that the model has been trained on a data set consisting of text and information available up until that point. Therefore, any events, advancements, or changes in various fields that have occurred since September 2021 will not be known to ChatGPT-4. Additionally, it should be noted that ChatGPT-4, like any AI language model, reflects the data on which it has been trained. As a result, its knowledge might contain inaccuracies, biases, or outdated information even for events and topics within its known time frame.

#### Microsoft Bing

The Microsoft Bing AI chatbot [7] is an intelligent conversational agent developed by Microsoft Corporation, designed to assist users in navigating the Microsoft Bing search engine and answering various queries. Leveraging AI, natural language processing, and machine learning, the Microsoft Bing AI chatbot understands user inputs and provides relevant information or search results accordingly. Integrated seamlessly with the Microsoft Bings platform, the chatbot offers a user-friendly and interactive way to engage with search functionalities, enhancing the overall user experience.

### Prompt Design

In the context of AI, especially with large language models, a “prompt” refers to a set of instructions or a question given to the AI to guide its response. The purpose of a prompt is to set clear expectations for the AI’s output and to ensure that the response generated aligns with the user’s intent.

A prompt was designed to secure the AI models’ ability to understand and generate accurate, relevant, and coherent responses in a formal and professional tone. Each prompt provided the AI models with the context of an academic paper and set the tone and expectations for the responses. The following specific prompt was used to ensure that both ChatGPT-4 and Microsoft Bing were primed for the task at hand:

I need your help with an academic paper. Please provide me with clear and concise explanations, using evidence and logical reasoning to support your responses. Your tone should be formal and professional, and your language should be free from errors and ambiguity. I am looking for accurate and well-supported information that will help me to achieve my academic goals.

### Data Collection

The interview with the 2 models took place on March 9, 2023, with early access to ChatGPT-4. Both ChatGPT-4 and Microsoft Bing were asked to provide an outline for a discussion article on the chosen topic, encompassing various aspects of general practice. This approach aimed to evaluate the AI models’ ability to synthesize information and structure a coherent, well-organized outline that could serve as a foundation for a comprehensive discussion article. As differences between the outlines are likely, the most comprehensive outline was used to ensure a meaningful comparison between interviews. The length of each question was limited to ensure accuracy and reduce the risk of errors during the conversation.

### Evaluation Criteria

It is important to note that the evaluation was conducted solely by one author, and the assessments were largely based on their subjective judgment. To compare and assess the quality of the AI-generated responses, the following evaluation criteria were established:

- Relevance: the extent to which the AI-generated response addresses the prompt and provides information pertinent to the question or topic.
- Accuracy: the degree to which the information provided is factually correct and up to date, based on the current state of knowledge in the field.
- Clarity: the clarity and coherence of the AI-generated response, including the logical flow of ideas and the use of precise, unambiguous language.
- Tone and style: the appropriateness of the tone and style of the AI-generated response, considering the formal and professional context of an academic paper.

To evaluate the evaluation criteria, a comprehensive literature search was conducted to identify areas where AI might be useful and implemented in general practice.

### Analysis

Each AI-generated response was analyzed independently, using the evaluation criteria, providing the strengths and weaknesses of each model. Hereafter, a comparison between the 2 models was conducted to establish differences. The results of the evaluation and comparison between the 2 models were then compiled and analyzed to determine the overall performance of ChatGPT-4 and Microsoft Bing related to the area of AI use in general practice and the areas preidentified, aiming at identifying the strengths and weaknesses of each AI model as well as any potential areas for improvement.

## Results

For a complete comparison, the full conversation with both ChatGPT-4 and Microsoft Bing models can be found in Multimedia Appendix 1.

### Relevance

#### Chat-GPT

GPT-4 offers a detailed analysis of AI applications in health care, focusing on general practice, its limitations, ethical concerns, and the importance of collaboration between AI and health care professionals. It provides comprehensive information, citing academic sources and studies, discussing AI algorithms, natural language processing, pattern recognition, evidence-based medicine, and personalized treatment plans. ChatGPT-4 also addresses data privacy, security concerns, and technical challenges while emphasizing the need to integrate AI systems with clinical workflows and patient needs. It provides a relevant and comprehensive examination of AI’s potential benefits and challenges in health care, emphasizing the need for integration with clinical workflows and a balanced approach to ensure optimal patient care.

#### Microsoft Bing

Microsoft Bing offers a brief overview of AI in general practice, addressing advantages and limitations without delving into specific applications or ethical considerations. It lacks the depth and citations and does not emphasize the importance of collaboration between AI and health care professionals. Although Microsoft Bing touches on themes that are relevant, it provides neither specific study references nor in-depth explanations, offering a more concise perspective (Table 1).

**Table 1:** Comparison of ChatGPT and Microsoft Bing in terms of topic relevance.

Evaluation criteria	ChatGPT-4	Microsoft Bing
Relevance	A detailed analysis of AIa applications in healthcare Comprehensive information and citing academic sources Emphasizing the need for integration with clinical workflows and a balanced approach to ensure optimal patient care	A brief overview of AI in general practice Lack of in-depth or specific study citations Offering a more concise perspective

^a^AI: artificial intelligence.

### Accuracy

#### ChatGPT

ChatGPT-4 included 23 of 32 (72%) precise peer-reviewed articles with high accuracy. The introduction and applications in general practice were 100% correct. However, it also cited 9 nonexistent articles, with 4 out of 7 inaccuracies in limitations and all 4 ethical considerations being inaccurately cited.

#### Microsoft Bing

Microsoft Bing included 6 of 13 (46%) highly accurate, peer-reviewed articles, along with 7 non–peer-reviewed but highly relevant articles. Ethical considerations and applications in general practice cited 3 and 2 non–peer-reviewed articles, respectively (Table 2).

The references provided from both models, along with the accuracy distribution, can be found in Multimedia Appendix 2.

**Table 2:** Comparison of ChatGPT and Microsoft Bing in terms of accuracy.

Evaluation criteria	ChatGPT-4	Microsoft Bing
Accuracy	A total of 23 out of 32 (72%) precise peer-reviewed articles, with high accuracy A total of 9 nonexistent articles, with specific inaccuracies	A total of 6 out of 13 (46%) highly accurate, peer-reviewed articles A total of 7 non–peer-reviewed but highly relevant articles

### Clarity

#### Chat GPT-4

Overall, the text generated by ChatGPT demonstrates a high level of clarity and coherence, exhibiting a logical flow of ideas and the use of precise, unambiguous language. The text is easy to follow and understand, even for readers who may not be familiar with the technical terms and concepts discussed.

#### Microsoft Bing

Similar to ChatGPT, the text exhibits a high level of clarity and coherence, with a logical flow of ideas and the use of precise, unambiguous language. It is easily comprehensible, even for readers unfamiliar with the technical terms and concepts discussed. However, the text could be improved by providing more details and examples to support the points made, as many areas are discussed in a more general manner (Table 3).

**Table 3:** Comparison of ChatGPT and Microsoft Bing in terms of clarity.

Evaluation Criteria	ChatGPT-4	Microsoft Bing
Clarity	The text is clear, coherent, and easy to understand, even for nontechnical readers.	The text is clear and coherent but could benefit from more detailed examples.

### Tone (Chat GPT-4 and Microsoft Bing)

Overall, the tone and style of the text are appropriate for the formal and professional context of an academic paper, effectively conveying complex ideas in a clear and objective manner (Table 4).

**Table 4:** Comparison of ChatGPT and Microsoft Bing in terms of tone.

Evaluation criteria	ChatGPT-4	Microsoft Bing
Tone	Appropriate for an academic paper, conveying ideas clearly and objectively	Appropriate for an academic paper, conveying ideas clearly and objectively

## Discussion

### Principal Findings

In recent years, AI has become an increasingly prevalent tool in various domains, including health care and academic research. AI language models, such as ChatGPT-4 and Microsoft Bing, have demonstrated the potential to assist researchers in generating and organizing content for academic papers. In the context of general practice, a rapidly evolving field with a growing need for accurate and relevant information, understanding the strengths and limitations of these AI models is crucial for researchers and practitioners alike. This paper aimed to compare and analyze the performance of ChatGPT-4 and Microsoft Bing in assisting with an academic paper in general practice, focusing on their relevance, accuracy, clarity, as well as tone and style. By examining their respective contributions and limitations, we seek to provide insights into their potential uses and areas for improvement in AI-assisted research.

In terms of relevance, ChatGPT-4 provided a detailed analysis of AI applications in health care, emphasizing the importance of collaboration between AI and health care professionals, while Microsoft Bing offered a concise overview without delving into specific applications or ethical considerations. As for accuracy, ChatGPT-4 accurately cited 72% (23/32) of peer-reviewed articles, but it also inaccurately cited 9 nonexistent articles. Microsoft Bing, on the other hand, included 6 of 13 (46%) accurate peer-reviewed articles and 7 non–peer-reviewed but highly relevant articles.

Regarding clarity, both ChatGPT-4 and Microsoft Bing demonstrated high levels of clarity and coherence, presenting a logical flow of ideas with precise, unambiguous language. Nevertheless, Microsoft Bing could benefit from providing more details and examples to support its points, as certain areas were discussed in a more general manner. Lastly, in terms of tone and style, both AI models used an appropriate tone and style for the formal and professional context of an academic paper, effectively conveying complex ideas in a clear and objective manner.

### Comparison With the Existing Literature

The results of this study, which compared the performance of ChatGPT-4 and Microsoft Bing in assisting with an academic paper in general practice, can be contextualized within the broader landscape of AI applications in health care and general practice research. The findings align with several previous studies that have highlighted the potential of AI language models, such as ChatGPT-4, to deliver relevant, detailed, and coherent information on complex subjects like health care [6,12].

The superior performance of ChatGPT-4 in providing comprehensive and in-depth analysis aligns with its advanced architecture and extensive training on a vast data set, which has been documented to enable the model to generate human-like text and engage in interactive conversations with users [12]. Similarly, the results are consistent with previous research that has emphasized the importance of collaboration between AI and health care professionals to achieve optimal patient care [13].

However, the observed weaknesses in ChatGPT-4’s accuracy, specifically in citing nonexistent articles, highlight the limitations of AI language models in some areas of academic research. This issue has been acknowledged in existing literature, where concerns have been raised about the potential for AI-generated content to include inaccuracies, biases, or misinformation [14].

In contrast, Microsoft Bing’s more concise approach to providing information echoes its primary function as a search engine assistant rather than a specialized AI language model. This result is consistent with the notion that AI chatbots, while capable of providing relevant information, may not always deliver the depth and detail required for more demanding academic tasks [15].

### Strengths

This study has some strengths, as follows:

- Prompt design: the study used a well-crafted prompt to ensure that both ChatGPT-4 and Microsoft Bing were primed for the task, which helped in generating accurate, relevant, and coherent responses in a formal and professional tone.
- Evaluation criteria: the established evaluation criteria (relevance, accuracy, clarity, as well as tone and style) provided a comprehensive framework for comparing and assessing the quality of the AI-generated responses.
- Analysis: the independent analysis of each AI-generated response, followed by a comparison between the 2 models, allowed for a thorough understanding of the strengths and weaknesses of each AI model.

### Weaknesses

The weaknesses of the study are the following:

- Data collection: the study’s data collection method, which involved interviewing the 2 models, may have been limited in scope. A more comprehensive approach involving a larger sample of questions or topics could have provided a broader understanding of the AI models’ capabilities.
- Knowledge cut-off: ChatGPT-4 has a knowledge cut-off date of September 2021, which may have limited its ability to provide up-to-date information in some instances.
- Limited exploration of AI models: the study only compared 2 AI models—ChatGPT-4 and Microsoft Bing. This may not provide a complete picture of the landscape of AI tools available for assisting with academic papers in general practice. Including more AI models, such as Google’s chatbot—Bard, in the comparison could have yielded a more comprehensive analysis. However, this model is not currently available in Denmark.

The strengths and weaknesses of each model are presented in Table 5.

**Table 5:** A side-by-side comparison of the features and aspects of ChatGPT-4 and Microsoft Bing’s artificial intelligence (AI) chatbot.

Feature or aspect	ChatGPT-4	Microsoft Bing
Developer	OpenAI	Microsoft Corporation
Primary function	Generating human-like text and engaging in interactive conversations	Assisting users in navigating the Microsoft Bing search engine and answering queries
Training or technology	Vast data set, context understanding, language, and reasoning abilities	Artificial intelligence, natural language processing, and machine learning
Special features	Answering questions, providing recommendations, and generating content	Integrating with the Bing platform and enhancing the search experience
Conversation limits	25 conversations per 3 hours	Limited to 20 prompts
Internet access	No	Yes
Knowledge cut-off	Up to 2021	Uses OpenAI technology with access to the internet and thus can acquire the newest information
Memory constraints	Forgets information within longer conversations and might stop midsentence in lengthy responses	Closely related to ChatGPT-4 in this area
Additional information	Some responses may require user prompts to be complete	Offers a user-friendly and interactive way to engage with search functionalities

### Implications for AI-Assisted Research

The findings of this study have several implications for researchers and practitioners using AI in general practice and other academic fields. These implications are as follows:

- Quality of AI-generated content: the comparison between ChatGPT-4 and Microsoft Bing demonstrates that the quality of AI-generated content can vary between models. Researchers and practitioners should be aware of the strengths and weaknesses of different AI models when selecting a tool to assist with their work.
- Importance of collaboration: both ChatGPT-4 and Microsoft Bing highlight the importance of collaboration between AI and health care professionals. AI systems should be designed to complement human expertise and foster collaboration, enhancing the overall quality of research and practice.
- Relevance and accuracy: ensuring the relevance and accuracy of AI-generated responses is crucial for researchers and practitioners. Although AI models can provide valuable insights, they might also generate inaccuracies or outdated information. Users must verify the information provided by AI models and cross-check it with up-to-date, reliable sources.
- Clarity and tone: AI-generated content should be clear and coherent; it should maintain an appropriate tone and style for the intended audience. Although AI models like ChatGPT-4 and Microsoft Bing show promising results in these aspects, users should carefully review and edit the generated content to ensure it meets the required standards.
- Ethical considerations: as AI continues to be integrated into various aspects of research and practice, ethical considerations must be addressed. Data privacy, security, and responsible use of AI-generated content are crucial to ensuring that AI is used responsibly and effectively in general practice and other academic fields.

Overall, the findings of this study indicate that AI models, such as ChatGPT-4 and Microsoft Bing, can provide valuable assistance in general practice and other academic fields. However, researchers and practitioners should be aware of the limitations and potential pitfalls of AI-generated content and use these tools thoughtfully and responsibly.

### Areas for Improvement and Future Research

#### AI Model Improvements

##### ChatGPT-4

Although ChatGPT-4 demonstrates strong performance in relevance, clarity, and tone, there is room for improvement in terms of accuracy, especially in relation to citing nonexistent articles. Enhancing the fact-checking and source validation capabilities of the model could help address this issue.

##### Microsoft Bing

Microsoft Bing could benefit from improvements in providing more in-depth, relevant content with proper citations. Enhancing the model’s understanding of specific academic contexts and ethical considerations would allow it to provide more comprehensive and valuable insights to the users.

#### Methodology Improvements

The methodology improvements required are as follows:

- Expanding the sample size: including more AI models in the comparison would provide a broader understanding of the capabilities and limitations of AI-assisted research.
- Diversifying the topics: evaluating AI-generated responses across a wider range of topics and academic fields could offer more generalizable insights into the strengths and weaknesses of AI-assisted research.
- Including human evaluation: adding a panel of human evaluators to assess the AI-generated content could help provide a more nuanced understanding of the quality and relevance of the responses.

#### Future Research Directions

Some directions for future research are explained below:

- Longitudinal studies: investigating the evolution of AI models over time, as they are updated and trained on new data, could provide valuable insights into the progress of AI-assisted research and the potential of these tools in various academic fields.
- Ethical implications: examining the ethical implications of AI-generated content in academic research, such as issues related to plagiarism, data privacy, and potential biases, could help develop best practices and guidelines for responsible use of AI in research.
- Integration with research workflows: exploring how AI models can be effectively integrated into existing research workflows and practices and identifying the most effective ways to combine AI-generated content with human expertise would help maximize the benefits of AI-assisted research.

By addressing these areas for improvement and exploring future research directions, researchers and practitioners can continue to refine the use of AI models in general practice and other academic fields, ultimately enhancing the quality, efficiency, and impact of their work.

### Conclusions

Our study comparing ChatGPT-4 and Microsoft Bing in assisting with writing an academic paper in general practice yielded several key findings. ChatGPT-4 demonstrated strong performance in terms of relevance, clarity, and tone, providing comprehensive information and detailed analysis of AI applications in health care. However, it exhibited weaknesses in accuracy, particularly in citing nonexistent articles. Microsoft Bing offered a more concise perspective, touching on relevant themes but lacking depth and proper citations.

In terms of methods used, the study incorporated prompt design, data collection, evaluation criteria, and analysis of AI-generated responses. The strengths of these methods include the design of a prompt that effectively engaged both AI models and the establishment of clear evaluation criteria. However, there is room for improvement in the methodology, such as expanding the sample size, diversifying the topics, and including human evaluation.

When comparing ChatGPT-4 and Microsoft Bing, ChatGPT-4 emerged as a more capable AI model for assisting with an academic paper in general practice. It provided a more in-depth, relevant, and coherent analysis of the topic; however, improvements in accuracy, particularly in source validation, would further enhance its utility. On the other hand, Microsoft Bing could benefit from improvements in providing more comprehensive content and proper citations to better support academic research.

In conclusion, ChatGPT-4 and Microsoft Bing present distinct pros and cons in academic writing. ChatGPT-4 excels in relevance and depth, but both AI models require improvement. Merging their strengths can produce comprehensive answers from ChatGPT-4 and up-to-date references from Microsoft Bing.

Despite their impressive abilities, these tools currently cannot author articles independently in certain areas. As AI models advance and incorporate current references and critical thinking, they may eventually conduct and create research autonomously.

This study’s findings hold substantial implications for AI-assisted research across diverse fields, emphasizing areas for refinement and future research directions to optimize AI models in academia. To mitigate risks, researchers must adopt a critical approach, corroborate information from various sources, and stay aware of AI models’ limitations. This approach allows them to harness AI while preserving the integrity and rigor of their work.

## Appendix

Multimedia Appendix 1 Interview with models.

Multimedia Appendix 2 References provided by models and their relevance.

## Abbreviations

AI: artificial intelligence
LAMBADA: LAnguage Modeling Broadened to Account for Discourse Aspects
