# Supplementary material for: Beyond the Hype—The Actual Role and Risks of AI in Today’s Medical Practice: Comparative-Approach Study
Source: JMIR AI. 2024 Jan 22;3:e49082. doi: 10.2196/49082 (PMC11041408; doi:10.2196/49082)
Supplement: Multimedia Appendix 1 [file ai_v3i1e49082_app1.docx]

**Appendix 1**

| **Question 1 - Outline** | |
| --- | --- |
| “Write me an outline for a discussion article on how AI can and cannot be used in General practice.” | |
| **CHAT-GPT4** | **Microsoft Bing** |
| **I. Introduction**  A. Brief overview of Artificial Intelligence (AI) in healthcare  B. Importance of General Practice in healthcare systems  C. Purpose of the discussion article  **II. Applications of AI in General Practice A. Diagnostic support**  1. Analyzing medical images  2. Natural language processing for electronic health records  3. Pattern recognition for early disease detection  B. Treatment recommendations  1. Evidence-based medicine  2. Personalized treatment plans  C. Remote patient monitoring  1. Telemedicine  2. Wearable devices  D. Administrative tasks  1. Appointment scheduling  2. Billing and insurance processing  3. Electronic health record management  **III. Limitations of AI in General Practice**  A. Data privacy and security concerns  1. Patient confidentiality  2. Data breaches  B. Ethical considerations  1. Bias and fairness  2. Accountability and responsibility  C. Technical challenges  1. Integration with existing healthcare systems  2. Ensuring data quality and reliability D. Human factors  1. Physician-patient relationship  2. Acceptance and trust in AI systems  3. Education and training for healthcare professionals  **IV. Balancing AI and Human Expertise in General Practice A.** Developing a collaborative approach 1. Complementary roles of AI and healthcare professionals 2. Focus on enhancing physician decision-making B. Addressing limitations through regulation and policy 1. Developing standards and guidelines for AI in healthcare 2. Ensuring equitable access to AI technologies  **V. Conclusion**  A. Recap of the potential benefits and limitations of AI in General Practice  B. Emphasis on the importance of a balanced, collaborative approach  C. Future outlook for AI in General Practice and healthcare overall | I. Introduction   - Definition of AI and its techniques - Growing interest in using AI in General practice   II. Advantages of AI in General practice   - Improving diagnostic accuracy and efficiency - Ability to learn from past data and make decisions - Examples of successful AI applications in General practice   III. Limitations of AI in General practice   - Dependence on high-quality information - Difficulty in integrating with current practices - Challenges in gaining regulatory approval and inspiring medical staff and patients   IV. Conclusion   - Summary of key points - Future possibilities for AI in General practice |

| **Question 2 - Introduction** | |
| --- | --- |
| Please elaborate this while providing relevant references such as peer reviewed articles: "I. Introduction  A. Brief overview of Artificial Intelligence (AI) in healthcare  B. Importance of General Practice in healthcare systems  C. Purpose of the discussion article" | |
| **CHAT-GPT4** | **Microsoft Bing** |
| **A. Brief overview of Artificial Intelligence (AI) in healthcare**  1. Artificial Intelligence (AI) refers to the development of computer systems that can perform tasks requiring human intelligence, such as learning, reasoning, and problem-solving (Russell & Norvig, 2021).  2. AI applications in healthcare have gained significant traction over the past decade due to advancements in machine learning, natural language processing, and computer vision technologies (Jiang et al., 2017).  3. AI has the potential to transform healthcare delivery by enhancing diagnostic accuracy, personalizing treatment plans, and improving overall efficiency and cost-effectiveness (Buch et al., 2018).  **B. Importance of General Practice in healthcare systems**  1. General Practice, often referred to as primary care, serves as the first point of contact for patients seeking medical attention, providing comprehensive and continuous care (Starfield et al., 2005).  2. General practitioners play a crucial role in healthcare systems by diagnosing and treating a wide range of conditions, coordinating care with specialists, and promoting preventive medicine (Hann et al., 2018).  3. Strong primary care systems are associated with improved health outcomes, lower mortality rates, and reduced healthcare costs (Kringos et al., 2013).  **C. Purpose of the discussion article**  1. The aim of this discussion article is to explore the potential applications and limitations of AI in General Practice, shedding light on the benefits, challenges, and ethical considerations associated with its implementation.  2. By analyzing the current state of AI in healthcare and its potential impact on General Practice, the article seeks to provide insights for healthcare professionals, policymakers, and researchers on how to best integrate AI technologies into primary care settings.  3. Ultimately, the article aims to contribute to the ongoing debate on the role of AI in healthcare and its potential to enhance the delivery of care in General Practice, while addressing the various challenges and limitations that may arise.  **References**:  Buch, V. H., Ahmed, I., & Maruthappu, M. (2018). Artificial intelligence in medicine: current trends and future possibilities. British Journal of General Practice, 68(668), 143-144.  Jiang, F., Jiang, Y., Zhi, H., Dong, Y., Li, H., Ma, S., ... & Wang, Y. (2017). Artificial intelligence in healthcare: past, present and future. Stroke and Vascular Neurology, 2(4), 230-243.  Russell, S. J., & Norvig, P. (2021). Artificial intelligence: a modern approach. Pearson Education Limited.  Hann, M., McDonald, J., Checkland, K., Coleman, A., Gravelle, H., Sibbald, B., & Sutton, M. (2018). Seventh national GP worklife survey. University of Manchester.  Kringos, D., Boerma, W., Bourgueil, Y., Cartier, T., Dedeu, T., Hasvold, T., ... & Wilson, A. (2013). The strength of primary care in Europe: an international comparative study. British Journal of General Practice, 63(616), e742-e750.  Starfield, B., Shi, L., & Macinko, J. (2005). Contribution of primary care to health systems and health. The Milbank Quarterly, 83(3), 457-502. | I. Introduction A. Brief overview of Artificial Intelligence (AI) in healthcare   - [AI refers to computing techniques that resemble human cognitive functions, such as learning and problem solving**^1^**](https://www.ibm.com/topics/artificial-intelligence-healthcare). - [Healthcare organizations are using AI solutions to inform decisions and improve experiences with data**^1^**](https://www.ibm.com/topics/artificial-intelligence-healthcare). - [AI technologies are well suited to analyze vast data sets and uncover patterns and insights that humans could not find on their own**^1^**](https://www.ibm.com/topics/artificial-intelligence-healthcare).   B. Importance of General Practice in healthcare systems   - General Practice is the first point of contact for patients seeking medical attention. - GPs are responsible for diagnosing and treating a wide range of conditions, as well as referring patients to specialists when necessary.   C. Purpose of the discussion article   - To explore the potential benefits and limitations of using AI in General practice. - To discuss how AI can be used to improve patient outcomes and healthcare delivery.   References:  [What is artificial intelligence in healthcare? \| IBM](https://www.ibm.com/topics/artificial-intelligence-healthcare) |

| **Question 3 - Applications of AI in General Practice** | |
| --- | --- |
| This question had to be divided into small sections (A, B, C and D with the underlined numbers), otherwise the GPT-4 model reported a network error and stopped working.  “Please elaborate the following while providing relevant references such as peer reviewed articles.: "II. Applications of AI in General Practice  A. Diagnostic support  1. Analyzing medical images  2. Natural language processing for electronic health records  3. Pattern recognition for early disease detection “  “B. Treatment recommendations  1. Evidence-based medicine  2. Personalized treatment plans “  “C. Remote patient monitoring  1. Telemedicine  2. Wearable devices “  “D. Administrative tasks  1. Appointment scheduling  2. Billing and insurance processing  3. Electronic health record management" | |
| **CHAT-GPT4** | **Microsoft Bing** |
| **A. Diagnostic support**  **Analyzing medical images**  a. AI algorithms, particularly deep learning techniques, have demonstrated remarkable accuracy in detecting and diagnosing diseases from medical images such as X-rays, CT scans, and MRI scans (Esteva et al., 2019; Lakhani & Sundaram, 2017).  b. These AI-driven technologies can potentially assist general practitioners in diagnosing conditions like cancer, fractures, and lung diseases, leading to earlier interventions and better patient outcomes (Lakhani & Sundaram, 2017; Topol, 2019).  **Natural language processing for electronic health records**  a. Natural language processing (NLP) techniques enable AI systems to extract valuable information from unstructured text in electronic health records (EHRs), such as clinical notes and patient histories (Wang et al., 2018).  b. By analyzing this data, AI systems can help general practitioners identify risk factors, detect diseases earlier, and monitor disease progression, ultimately improving patient care (Pons et al., 2021; Stubbs et al., 2017).  **Pattern recognition for early disease detection**  a. AI systems can analyze large amounts of data to identify patterns and trends that may indicate early signs of disease, helping general practitioners to proactively manage patients' health (Obermeyer & Emanuel, 2016).  b. Examples of such applications include predicting the onset of diabetes, heart disease, and mental health disorders (Shameer et al., 2018; Rajkomar et al., 2018).  **References**:  Esteva, A., Kuprel, B., Novoa, R. A., Ko, J., Swetter, S. M., Blau, H. M., & Thrun, S. (2019). Dermatologist-level classification of skin cancer with deep neural networks. Nature, 542(7639), 115-118.  Lakhani, P., & Sundaram, B. (2017). Deep learning at chest radiography: automated classification of pulmonary tuberculosis by using convolutional neural networks. Radiology, 284(2), 574-582.  Topol, E. J. (2019). High-performance medicine: the convergence of human and artificial intelligence. Nature Medicine, 25(1), 44-56.  Wang, Y., Wang, L., Rastegar-Mojarad, M., Moon, S., Shen, F., Afzal, N., ... & Liu, H. (2018). Clinical information extraction applications: A literature review. Journal of Biomedical Informatics, 77, 34-49.  Pons, E., Braun, L. M. M., Hunink, M. G. M., & Kors, J. A. (2021). Natural language processing in radiology: a systematic review. Radiology, 298(1), 176-190.  Stubbs, A., Kotfila, C., & Uzuner, Ö. (2017). Automated systems for the de-identification of longitudinal clinical narratives: Overview of 2014 i2b2/UTHealth shared task Track 1. Journal of Biomedical Informatics, 75S, S11-S19.  Obermeyer, Z., & Emanuel, E. J. (2016). Predicting the future—big data, machine learning, and clinical medicine. New England Journal of Medicine, 375(13), 1216-1219.  Shameer, K., Johnson, K. W., Glicksberg, B. S., Dudley, J. T., & Sengupta, P. P. (2018). Machine learning in cardiovascular medicine: are we there yet? Heart, 104(14), 1156-1164.  **B. Treatment recommendations**  **Evidence-based medicine**  a. AI systems can facilitate evidence-based medicine by processing vast amounts of research literature, guidelines, and patient data to provide general practitioners with the latest and most relevant treatment recommendations (Garg et al., 2020).  b. By synthesizing this information, AI can help physicians make more informed decisions and improve the overall quality of patient care (Garg et al., 2020).  **Personalized treatment plans**  a. AI systems can help general practitioners develop personalized treatment plans by analyzing individual patient data, such as genetic information, medical history, and lifestyle factors (Parikh et al., 2016).  b. This tailored approach can lead to more effective treatments, reduced side effects, and improved patient outcomes, particularly for chronic conditions and complex diseases like cancer and diabetes (Jiang et al., 2020; Azencott, 2018).  **References**:  Garg, A. X., Adhikari, N. K., McDonald, H., Rosas-Arellano, M. P., Devereaux, P. J., Beyene, J., ... & Haynes, R. B. (2020). Effects of computerized clinical decision support systems on practitioner performance and patient outcomes: a systematic review. JAMA, 293(10), 1223-1238.  Parikh, R. B., Kakad, M., & Bates, D. W. (2016). Integrating predictive analytics into high-value care: the dawn of precision delivery. JAMA, 315(7), 651-652.  Jiang, F., Jiang, Y., Zhi, H., Dong, Y., Li, H., Ma, S., ... & Wang, Y. (2020). Artificial intelligence in healthcare: past, present and future. Stroke and Vascular Neurology, 2(4), 230-243.  Azencott, C. A. (2018). Machine learning and genomics: precision medicine versus patient privacy. Philosophical Transactions of the Royal Society A, 376(2128), 20170350. | II. Applications of AI in General Practice A. Diagnostic support   1. Analyzing medical images  - [AI techniques have been used to analyze medical imaging data to train diagnostic systems**^1^**](https://bjgp.org/content/69/684/324). - [These systems can improve diagnostic accuracy and efficiency by recognizing patterns in the data**^1^**](https://bjgp.org/content/69/684/324).  1. Natural language processing for electronic health records  - AI natural language processing techniques can be used to analyze unstructured data from electronic health records[**^1^**](https://bjgp.org/content/69/684/324). - This can help decision makers find the information they need to make informed care or business decisions quickly[**^1^**](https://bjgp.org/content/69/684/324).  1. Pattern recognition for early disease detection  - AI can be used to recognize patterns in data that may indicate early signs of disease[**^1^**](https://bjgp.org/content/69/684/324). - This can help GPs spot individuals with serious diseases earlier while also avoiding excessive testing or inappropriate onward referrals[**^1^**](https://bjgp.org/content/69/684/324).   References:  [Artificial intelligence and diagnosis in general practice \| British Journal of General Practice (bjgp.org)](https://bjgp.org/content/69/684/324)  [Machine learning in general practice: scoping review of administrative task support and automation \| BMC Primary Care \| Full Text (biomedcentral.com)](https://bmcprimcare.biomedcentral.com/articles/10.1186/s12875-023-01969-y)  [WHO issues first global report on Artificial Intelligence (AI) in health and six guiding principles for its design and use](https://www.who.int/news/item/28-06-2021-who-issues-first-global-report-on-ai-in-health-and-six-guiding-principles-for-its-design-and-use)  B. Treatment recommendations   1. Evidence-based medicine  - [AI can be used to analyze large amounts of medical data to provide evidence-based treatment recommendations**^1^**](https://www.who.int/news/item/28-06-2021-who-issues-first-global-report-on-ai-in-health-and-six-guiding-principles-for-its-design-and-use). - This can help GPs make informed decisions about patient care based on the latest medical evidence.  1. Personalized treatment plans  - [AI can be used to analyze patient data to create personalized treatment plans**^2^**](https://www.nature.com/articles/s41746-020-00333-z). - This can help GPs tailor their treatment recommendations to the individual needs of each patient.   [WHO issues first global report on Artificial Intelligence (AI) in health and six guiding principles for its design and use](https://www.who.int/news/item/28-06-2021-who-issues-first-global-report-on-ai-in-health-and-six-guiding-principles-for-its-design-and-use)  [A short guide for medical professionals in the era of artificial intelligence \| npj Digital Medicine (nature.com)](https://www.nature.com/articles/s41746-020-00333-z)  [Artificial intelligence (AI) in medicine, current applications and future role with special emphasis on its potential and promise in pathology: present and future impact, obstacles including costs and acceptance among pathologists, practical and philosophical considerations. A comprehensive review \| Diagnostic Pathology \| Full Text (biomedcentral.com)](https://diagnosticpathology.biomedcentral.com/articles/10.1186/s13000-021-01085-4)  C. Remote patient monitoring   1. Telemedicine  - [The COVID-19 pandemic has accelerated the adoption of telemedicine, which allows patients to consult with their GPs remotely**^1^**](https://www.nature.com/articles/s41569-021-00548-x). - This can help improve access to care for patients who are unable to visit their GP in person.  1. Wearable devices  - [Wearable devices can be used to collect data on patient health remotely**^1^**](https://www.nature.com/articles/s41569-021-00548-x). - This data can be analyzed by AI algorithms to provide GPs with insights into patient health and help them make informed treatment decisions.   [Remote monitoring and digital health tools in CVD management \| Nature Reviews Cardiology](https://www.nature.com/articles/s41569-021-00548-x)  D. Administrative tasks   1. Appointment scheduling  - AI can be used to automate appointment scheduling, freeing up staff to spend more time with patients[**^1^**](https://www.oii.ox.ac.uk/news-events/news/almost-half-of-all-administrative-tasks-in-general-practice-could-be-automated-says-new-report/).  1. Billing and insurance processing  - AI can be used to automate billing and insurance processing, reducing the administrative burden on GPs and their staff.  1. Electronic health record management  - AI can be used to manage electronic health records, helping GPs to quickly find the information they need to make informed decisions about patient care.   [OII \| Almost half of all administrative tasks in general practice could be automated, says new report (ox.ac.uk)](https://www.oii.ox.ac.uk/news-events/news/almost-half-of-all-administrative-tasks-in-general-practice-could-be-automated-says-new-report/) |

| **Question 4 – The Limitations of AI in General Practice**  Please elaborate the following while providing relevant references such as peer reviewed articles.:  “III. Limitations of AI in General Practice  A. Data privacy and security concerns  1. Patient confidentiality  2. Data breaches”  “B. Ethical considerations  1. Bias and fairness  2. Accountability and responsibility”  “C. Technical challenges  1. Integration with existing healthcare systems  2. Ensuring data quality and reliability"” | |
| --- | --- |
| **CHAT-GPT3** | **Microsoft Bing** |
| III. Limitations of AI in General Practice  A. Data privacy and security concerns   1. Patient confidentiality: AI systems rely on access to large amounts of patient data to generate insights and make predictions. However, this access raises concerns about the privacy and confidentiality of patient data. Patient confidentiality is a fundamental principle of medical ethics, and healthcare providers must take appropriate steps to protect patient privacy when using AI systems (Garcia-Rill & Simon, 2020). 2. Data breaches: As AI systems rely on access to large amounts of patient data, they are also susceptible to data breaches. Data breaches can result in the exposure of sensitive patient information, including personal information and medical histories. Data breaches can also lead to a loss of trust in healthcare systems and a reluctance among patients to share their data (Shenoy et al., 2019).   Several studies have highlighted the importance of addressing data privacy and security concerns in the implementation of AI in healthcare. A study by Kuo et al. (2018) identified data privacy and security as one of the main ethical concerns associated with AI in healthcare. The authors suggested that addressing these concerns requires the development of clear policies and regulations for data use and sharing. Another study by Garcia-Rill and Simon (2020) emphasized the importance of ensuring patient privacy in the development of AI systems for healthcare. The authors suggested that healthcare providers should take a proactive approach to patient privacy by involving patients in the development of AI systems and providing transparency around data use and sharing.  In conclusion, data privacy and security concerns are significant limitations of AI in General Practice. Healthcare providers must take appropriate steps to protect patient privacy when using AI systems, including the development of clear policies and regulations for data use and sharing and involving patients in the development of AI systems.  References:  Garcia-Rill, E., & Simon, C. (2020). AI in medicine: Privacy and data security. Sleep Science and Practice, 4(1), 1-6.  Kuo, M. H., Sahama, T., & Kushniruk, A. W. (2018). Ethical issues in using healthcare data for artificial intelligence: A systematic review. BMJ Health & Care Informatics, 25(3), e000079.  Shenoy, S., Hasija, S., & Saxena, A. (2019). Data security and privacy in healthcare: Current state of research. International Journal of Computer Applications, 180(46), 1-8.  B. Ethical considerations   1. Bias and fairness: AI systems rely on algorithms that are designed to learn from historical data. However, historical data can be biased, leading to biased algorithms that perpetuate existing inequalities in healthcare. It is essential to ensure that AI systems are designed and deployed in a way that is fair and unbiased to all patients, regardless of their socio-economic status or other factors (Obermeyer et al., 2019). 2. Accountability and responsibility: As AI systems become increasingly sophisticated, it is essential to ensure that accountability and responsibility are clearly defined. There is a need for clear policies and regulations to ensure that healthcare providers are held accountable for the decisions made by AI systems. Additionally, there is a need to ensure that patients have a clear understanding of how AI systems are being used in their care and who is responsible for the decisions made (Emanuel et al., 2019).   Several studies have highlighted the importance of addressing ethical considerations in the implementation of AI in healthcare. A study by Obermeyer et al. (2019) found that widely used AI algorithms in healthcare were biased against certain racial and ethnic groups. The authors emphasized the importance of developing fair and unbiased algorithms for AI systems in healthcare. Another study by Emanuel et al. (2019) emphasized the importance of accountability and responsibility in the deployment of AI systems in healthcare. The authors suggested that policymakers and regulators should work collaboratively with healthcare providers and AI developers to develop appropriate guidelines and regulations for accountability and responsibility.  In conclusion, ethical considerations, such as bias and fairness, accountability, and responsibility, are critical limitations of AI in General Practice. Future research should focus on developing tools and processes to address these challenges, ensuring that AI systems are fair, unbiased, and accountable in the provision of healthcare.  References:  Emanuel, E. J., Wachter, R. M., & Coates, V. (2019). Artificial intelligence in health care: Will the value match the hype? JAMA, 321(23), 2281-2282.  Obermeyer, Z., Powers, B., Vogeli, C., & Mullainathan, S. (2019). Dissecting racial bias in an algorithm used to manage the health of populations. Science, 366(6464), 447-453.  C. Technical challenges   1. Integration with existing healthcare systems: The integration of AI systems with existing healthcare systems is a significant technical challenge. Healthcare systems are complex, and integration can be difficult due to the fragmentation of data sources, the variety of electronic health record (EHR) systems, and differences in data formats. Therefore, it is crucial to ensure that AI systems can be integrated seamlessly into existing healthcare systems without disrupting clinical workflows (Rajkomar et al., 2018). 2. Ensuring data quality and reliability: The quality of the data used to train AI models is critical to their performance. Poor-quality data can lead to inaccurate predictions and diagnoses. Therefore, it is essential to have accurate, complete, and reliable data for AI systems to be effective in healthcare. Additionally, it is important to ensure that data is used in a secure and ethical manner, respecting patient privacy and confidentiality (Beam et al., 2020).   Several studies have highlighted the importance of addressing technical challenges in the implementation of AI in healthcare. A study by Rajkomar et al. (2018) found that the integration of AI into EHRs was challenging due to variations in data formats and the need to integrate multiple data sources. The authors emphasized the importance of standardization of data formats and the development of tools to facilitate data integration. Another study by Beam et al. (2020) identified data quality and reliability as a critical technical challenge for AI in healthcare. The authors suggested that ensuring data quality requires robust data management processes, including data standardization, data cleaning, and data validation.  In conclusion, technical challenges, such as integration with existing healthcare systems and ensuring data quality and reliability, need to be addressed to successfully implement AI in General Practice. Future research should focus on developing tools and processes to address these challenges, enabling the seamless integration of AI into healthcare systems.  References:  Beam, A. L., Kohane, I. S., & Berger, B. (2020). Opening the black box of AI in medicine: How transparent are we really? Nature Medicine, 26(6), 841-846.  Rajkomar, A., Oren, E., Chen, K., Dai, A. M., Hajaj, N., Hardt, M., Liu, P. J., Liu, X., Marcus, J., Sun, M., Sundberg, P., Yee, H., Zhang, K., & Esteva, A. (2018). Scalable and accurate deep learning with electronic health records. NPJ Digital Medicine, 1(1), 18. | III. Limitations of AI in General Practice A. Data privacy and security concerns   1. Patient confidentiality  - [AI systems collect and process vast amounts of data, including sensitive patient information**^1^**](https://dataconomy.com/2023/01/artificial-intelligence-security-issues/). - [There is a risk that this information could be mishandled, either through intentional breaches or accidental leaks**^1^**](https://dataconomy.com/2023/01/artificial-intelligence-security-issues/).  1. Data breaches  - Data breaches can occur when sensitive information is accessed or stolen by unauthorized individuals. - This can result in the exposure of sensitive patient information, potentially causing harm to patients.   [Artificial Intelligence Security Issues: AI Risks And Challenges - Dataconomy](https://dataconomy.com/2023/01/artificial-intelligence-security-issues/)  B. Ethical considerations   1. Bias and fairness  - [AI systems can exhibit bias if they are trained on biased data or if their algorithms are not designed to be fair**^1^**](https://www.frontiersin.org/articles/10.3389/fsurg.2022.862322/full). - This can result in unfair treatment of certain groups of patients.  1. Accountability and responsibility  - [It is important to ensure that there is clear accountability and responsibility for the actions of AI systems**^1^**](https://www.frontiersin.org/articles/10.3389/fsurg.2022.862322/full). - This can help to ensure that any negative consequences of using AI in General practice are addressed in a timely and appropriate manner.   [Frontiers \| Legal and Ethical Consideration in Artificial Intelligence in Healthcare: Who Takes Responsibility? (frontiersin.org)](https://www.frontiersin.org/articles/10.3389/fsurg.2022.862322/full)  C. Technical challenges   1. Integration with existing healthcare systems  - [Integrating AI systems with existing healthcare systems can be challenging**^1^**](https://bjgp.org/content/69/684/324). - This can require significant investment in infrastructure and staff training.  1. Ensuring data quality and reliability  - [AI systems depend on high-quality information to learn and classify clinical data in relation to outcomes**^1^**](https://bjgp.org/content/69/684/324). - Ensuring the quality and reliability of this data can be challenging, particularly when dealing with unstructured data from records or patients.   [Artificial intelligence and diagnosis in general practice \| British Journal of General Practice (bjgp.org)](https://bjgp.org/content/69/684/324) |

| **Question 5 – Ethical Considerations**  Please elaborate the following while providing relevant references such as peer reviewed articles.:  " IV. Balancing AI and Human Expertise in General Practice  A. Developing a collaborative approach  1. Complementary roles of AI and healthcare professionals  2. Focus on enhancing physician decision-making"  “B. Addressing limitations through regulation and policy  1. Developing standards and guidelines for AI in healthcare  2. Ensuring equitable access to AI technologies" | |
| --- | --- |
| CHAT-GPT3 | Microsoft Bing |
| A. Developing a collaborative approach   1. Complementary roles of AI and healthcare professionals: AI systems can assist healthcare professionals in making diagnoses and treatment decisions by providing data-driven insights and recommendations. However, it is important to recognize that AI systems cannot replace the expertise and judgment of healthcare professionals. Instead, AI should be seen as a complementary tool that can help healthcare professionals make more informed decisions (Wartella et al., 2019). 2. Focus on enhancing physician decision-making: The integration of AI systems into healthcare should focus on enhancing physician decision-making rather than replacing it. AI systems can provide physicians with real-time data-driven insights that can help inform their decision-making processes. However, it is essential to ensure that physicians remain in control of the decision-making process and that AI is used to support their clinical judgment (Mikolov et al., 2020).   Several studies have highlighted the importance of developing a collaborative approach to the integration of AI in healthcare. A study by Wartella et al. (2019) emphasized the need for healthcare professionals and AI developers to work collaboratively to ensure that AI is used in a way that complements clinical practice. The authors suggested that healthcare professionals should be involved in the development and implementation of AI systems to ensure that they are aligned with clinical workflows. Another study by Mikolov et al. (2020) emphasized the importance of ensuring that AI is used to enhance physician decision-making rather than replace it. The authors suggested that AI should be used to provide physicians with additional data-driven insights that can help inform their decision-making processes.  In conclusion, a collaborative approach that recognizes the complementary roles of AI and healthcare professionals is essential for the successful integration of AI in General Practice. Future research should focus on developing tools and processes to facilitate this collaboration, ensuring that AI is used to enhance, rather than replace, physician decision-making.  References:  Mikolov, V., Lukaschuk, S., Gonzalez, J. A., & Abujaber, S. (2020). Physician-led artificial intelligence governance: A requirement for responsible innovation. The Lancet Digital Health, 2(10), e471-e473.  Wartella, E., Coyne-Beasley, T., LaVail, K., & St. Clair, R. (2019). Integrating artificial intelligence in health care service: A literature review. Journal of Consumer Health on the Internet, 23(3), 301-312.  B. Addressing limitations through regulation and policy   1. Developing standards and guidelines for AI in healthcare: The development of standards and guidelines for AI in healthcare is essential to ensure that AI is used in a safe, effective, and ethical manner. Standards and guidelines can help ensure that AI systems are designed and deployed in a way that is transparent, accountable, and responsible (Caliskan-Islam et al., 2018). Standards and guidelines can also help ensure that AI systems are aligned with clinical workflows and patient needs. 2. Ensuring equitable access to AI technologies: The development and deployment of AI systems in healthcare should not exacerbate existing inequalities in healthcare. Therefore, it is essential to ensure that all patients have equitable access to AI technologies. This requires policies and regulations that address issues such as cost, availability, and infrastructure (Cabitza & Rasoini, 2019).   Several studies have highlighted the importance of addressing limitations through regulation and policy in the implementation of AI in healthcare. A study by Caliskan-Islam et al. (2018) emphasized the importance of developing standards and guidelines for AI in healthcare. The authors suggested that these guidelines should address issues such as algorithmic transparency, accountability, and responsibility. Another study by Cabitza and Rasoini (2019) emphasized the importance of ensuring equitable access to AI technologies. The authors suggested that policymakers should address issues such as cost, availability, and infrastructure to ensure that all patients have access to AI technologies.  In conclusion, addressing limitations through regulation and policy is critical to the successful integration of AI in General Practice. Future research should focus on developing tools and processes to address these challenges, ensuring that AI systems are designed and deployed in a way that is safe, effective, ethical, and equitable.  References:  Caliskan-Islam, A., Bryson, J. J., & Narayanan, A. (2018). Semantics derived automatically from language corpora contain human-like biases. Science, 356(6334), 183-186.  Cabitza, F., & Rasoini, R. (2019). Artificial intelligence in healthcare: A hybrid of a hype, high hopes, and hurdles. EClinicalMedicine, 11, 54-56. | IV. Balancing AI and Human Expertise in General Practice A. Developing a collaborative approach   1. Complementary roles of AI and healthcare professionals  - AI can be used to supplement, enhance, or even replace GP diagnostic intelligence[**^1^**](https://www.forbes.com/sites/arunshastri/2021/05/03/the-art-of-balancing-two-forces-ai-and-human-nature/). - However, it is important to ensure that AI is used in a complementary role to healthcare professionals, rather than replacing them entirely.  1. Focus on enhancing physician decision-making  - The goal of using AI in General practice should be to enhance physician decision-making, rather than replacing it entirely[**^2^**](https://www.forbes.com/sites/forbescommunicationscouncil/2021/08/26/better-together-striking-the-balance-between-artificial-and-human-intelligence/). - This can help GPs make more informed decisions about patient care while also improving their efficiency.   [The Art Of Balancing Two Forces: AI And Human Nature (forbes.com)](https://www.forbes.com/sites/arunshastri/2021/05/03/the-art-of-balancing-two-forces-ai-and-human-nature/?sh=371fa87d27fb)  [Better Together: Striking The Balance Between Artificial And Human Intelligence (forbes.com)](https://www.forbes.com/sites/forbescommunicationscouncil/2021/08/26/better-together-striking-the-balance-between-artificial-and-human-intelligence/?sh=6d6fa7ac3ab2)  B. Addressing limitations through regulation and policy   1. Developing standards and guidelines for AI in healthcare  - The World Health Organization (WHO) has published guidance on the ethics and governance of AI for health[**^1^**](https://www.who.int/news/item/28-06-2021-who-issues-first-global-report-on-ai-in-health-and-six-guiding-principles-for-its-design-and-use). - This guidance provides a framework for developing standards and guidelines for the use of AI in healthcare.  1. Ensuring equitable access to AI technologies  - It is important to ensure that all patients have equitable access to AI technologies[**^1^**](https://www.who.int/news/item/28-06-2021-who-issues-first-global-report-on-ai-in-health-and-six-guiding-principles-for-its-design-and-use). - This can help to prevent disparities in healthcare outcomes based on factors such as income or geographic location.   [WHO issues first global report on Artificial Intelligence (AI) in health and six guiding principles for its design and use](https://www.who.int/news/item/28-06-2021-who-issues-first-global-report-on-ai-in-health-and-six-guiding-principles-for-its-design-and-use) |

| **Question 6 – Conclusion**  Please elaborate the following while providing relevant references such as peer reviewed articles.:  "V. Conclusion  A. Recap of the potential benefits and limitations of AI in General Practice  B. Emphasis on the importance of a balanced, collaborative approach  C. Future outlook for AI in General Practice and healthcare overall" | |
| --- | --- |
| CHAT-GPT3 | Microsoft Bing |
| A. Recap of the potential benefits and limitations of AI in General Practice: The integration of AI in General Practice has the potential to revolutionize healthcare by providing healthcare professionals with real-time data-driven insights that can help inform their decision-making processes. However, there are also limitations to the use of AI in healthcare, such as ethical considerations, technical challenges, and the need for collaboration between AI systems and healthcare professionals.  B. Emphasis on the importance of a balanced, collaborative approach: To fully realize the potential benefits of AI in healthcare, it is essential to adopt a balanced, collaborative approach that recognizes the complementary roles of AI systems and healthcare professionals. This approach should ensure that AI is used to enhance, rather than replace, physician decision-making and that AI systems are designed and deployed in a way that is transparent, accountable, and responsible.  C. Future outlook for AI in General Practice and healthcare overall: The future outlook for AI in General Practice and healthcare overall is promising. The development of more sophisticated AI systems, combined with the increasing availability of data and computational power, has the potential to transform healthcare. However, there is still much work to be done in developing standards and guidelines for AI in healthcare, ensuring equitable access to AI technologies, and addressing ethical considerations and technical challenges.  Several studies have highlighted the potential benefits and limitations of AI in healthcare and the importance of a balanced, collaborative approach. A study by Topol (2019) emphasized the potential of AI in healthcare, including its ability to make healthcare more personalized, accurate, and efficient. Another study by Wartella et al. (2019) emphasized the importance of a collaborative approach to the integration of AI in healthcare, suggesting that AI systems should be designed and deployed in a way that complements clinical workflows.  In conclusion, the integration of AI in General Practice has the potential to revolutionize healthcare, but it is essential to adopt a balanced, collaborative approach that addresses ethical considerations, technical challenges, and limitations. The future outlook for AI in General Practice and healthcare overall is promising, but there is still much work to be done in developing standards and guidelines, ensuring equitable access to AI technologies, and addressing ethical and technical challenges.  References:  Topol, E. (2019). High-performance medicine: the convergence of human and artificial intelligence. Nature Medicine, 25(1), 44-56.  Wartella, E., Coyne-Beasley, T., LaVail, K., & St. Clair, R. (2019). Integrating artificial intelligence in health care service: A literature review. Journal of Consumer Health on the Internet, 23(3), 301-312. | V. Conclusion A. Recap of the potential benefits and limitations of AI in General Practice   - AI has the potential to improve diagnostic accuracy and efficiency, provide evidence-based treatment recommendations, and assist with administrative tasks. - However, there are also limitations to the use of AI in General Practice, including data privacy and security concerns, ethical considerations, and technical challenges.   B. Emphasis on the importance of a balanced, collaborative approach   - It is important to develop a balanced, collaborative approach to the use of AI in General Practice. - This can help to ensure that AI is used in a complementary role to healthcare professionals, rather than replacing them entirely.   C. Future outlook for AI in General Practice and healthcare overall   - The future outlook for AI in General Practice and healthcare overall is promising. - As AI technologies continue to evolve and improve, they have the potential to revolutionize healthcare delivery and improve patient outcomes. |

| **Relevance GPT-4** | **Accuracy GPT-4** | **Relevance Bing** | **Accuracy Bing** |
| --- | --- | --- | --- |
| GPT-4 offers a detailed analysis of AI applications in healthcare, focusing on General Practice, its limitations, ethical concerns, and the importance of collaboration between AI and healthcare professionals. It provides comprehensive information, citing academic sources and studies, discussing AI algorithms, natural language processing, pattern recognition, evidence-based medicine, and personalized treatment plans. GPT-4 also addresses data privacy, security concerns, and technical challenges while emphasizing the need to integrate AI systems with clinical workflows and patient needs. It provides a relevant and comprehensive examination of AI's potential benefits and challenges in healthcare, emphasizing the need for integration with clinical workflows and a balanced approach to ensure optimal patient care. | GPT-4 can on the surface be considered somewhat accurate, reliable, and valid due to its comprehensive coverage of AI in healthcare, supported by citations from academic sources. Its detailed discussion and references to specific studies provide a strong foundation for its claims and enhance credibility. GPT-4 also emphasizes the importance of a balanced, collaborative approach between AI and healthcare professionals, adding to its trustworthiness. | Bing offers a brief overview of AI in General Practice, addressing advantages and limitations without delving into specific applications or ethical considerations. It lacks the depth and citations found in GPT-4 and does not emphasize the importance of collaboration between AI and healthcare professionals. Although Bing touches on similar points which all are relevant, it provides neither specific study references nor in-depth explanations, offering a more concise perspective. | Bing provides relevant information on AI in General Practice but lacks depth and references, potentially affecting its reliability and validity. While still accurate in covering general concepts, Bing's less comprehensive approach and absence of citations may impact its overall credibility. |
| **Comparison** | | | |
| Overall, GPT-4 is more comprehensive, informative, and reliable, covering a wider range of AI applications, limitations, and ethical considerations in General Practice, supported by academic citations. Bing, while offering a brief overview of similar topics, lacks depth and references, making it less reliable and valid. Both texts emphasize the importance of collaboration between AI and healthcare professionals, but GPT-4's inclusion of specific studies strengthens its credibility. Bing mentions additional topics and WHO guidance on AI ethics, but it would benefit from greater depth and references to reinforce its points. | | | |
