# Supplementary material for: Beyond the Hype—The Actual Role and Risks of AI in Today’s Medical Practice: Comparative-Approach Study
Source: JMIR AI. 2024 Jan 22;3:e49082. doi: 10.2196/49082 (PMC11041408; doi:10.2196/49082)
Supplement: Multimedia Appendix 2 [file ai_v3i1e49082_app2.docx]

**Appendix 2**

| **Chat GPT-4** | **Bing** |
| --- | --- |
| *Peer reviewed article & high accuracy* | |
| **Introduction:**  Buch, V. H., Ahmed, I., & Maruthappu, M. (2018). Artificial intelligence in medicine: current trends and future possibilities. British Journal of General Practice, 68(668), 143-144.  Jiang, F., Jiang, Y., Zhi, H., Dong, Y., Li, H., Ma, S., ... & Wang, Y. (2017). Artificial intelligence in healthcare: past, present and future. Stroke and Vascular Neurology, 2(4), 230-243.  Russell, S. J., & Norvig, P. (2021). Artificial intelligence: a modern approach. Pearson Education Limited.  Hann, M., McDonald, J., Checkland, K., Coleman, A., Gravelle, H., Sibbald, B., & Sutton, M. (2018). Seventh national GP worklife survey. University of Manchester.  Kringos, D., Boerma, W., Bourgueil, Y., Cartier, T., Dedeu, T., Hasvold, T., ... & Wilson, A. (2013). The strength of primary care in Europe: an international comparative study. British Journal of General Practice, 63(616), e742-e750.  Starfield, B., Shi, L., & Macinko, J. (2005). Contribution of primary care to health systems and health. The Milbank Quarterly, 83(3), 457-502.  **Applications of AI in General Practice**  Esteva, A., Kuprel, B., Novoa, R. A., Ko, J., Swetter, S. M., Blau, H. M., & Thrun, S. (2019). Dermatologist-level classification of skin cancer with deep neural networks. Nature, 542(7639), 115-118.  Lakhani, P., & Sundaram, B. (2017). Deep learning at chest radiography: automated classification of pulmonary tuberculosis by using convolutional neural networks. Radiology, 284(2), 574-582.  Topol, E. J. (2019). High-performance medicine: the convergence of human and artificial intelligence. Nature Medicine, 25(1), 44-56.  Wang, Y., Wang, L., Rastegar-Mojarad, M., Moon, S., Shen, F., Afzal, N., ... & Liu, H. (2018). Clinical information extraction applications: A literature review. Journal of Biomedical Informatics, 77, 34-49.  Pons, E., Braun, L. M. M., Hunink, M. G. M., & Kors, J. A. (2021). Natural language processing in radiology: a systematic review. Radiology, 298(1), 176-190.  Stubbs, A., Kotfila, C., & Uzuner, Ö. (2017). Automated systems for the de-identification of longitudinal clinical narratives: Overview of 2014 i2b2/UTHealth shared task Track 1. Journal of Biomedical Informatics, 75S, S11-S19.  Obermeyer, Z., & Emanuel, E. J. (2016). Predicting the future—big data, machine learning, and clinical medicine. New England Journal of Medicine, 375(13), 1216-1219.  Shameer, K., Johnson, K. W., Glicksberg, B. S., Dudley, J. T., & Sengupta, P. P. (2018). Machine learning in cardiovascular medicine: are we there yet? Heart, 104(14), 1156-1164.  Garg, A. X., Adhikari, N. K., McDonald, H., Rosas-Arellano, M. P., Devereaux, P. J., Beyene, J., ... & Haynes, R. B. (2020). Effects of computerized clinical decision support systems on practitioner performance and patient outcomes: a systematic review. JAMA, 293(10), 1223-1238.  Parikh, R. B., Kakad, M., & Bates, D. W. (2016). Integrating predictive analytics into high-value care: the dawn of precision delivery. JAMA, 315(7), 651-652.  Jiang, F., Jiang, Y., Zhi, H., Dong, Y., Li, H., Ma, S., ... & Wang, Y. (2020). Artificial intelligence in healthcare: past, present and future. Stroke and Vascular Neurology, 2(4), 230-243.  Azencott, C. A. (2018). Machine learning and genomics: precision medicine versus patient privacy. Philosophical Transactions of the Royal Society A, 376(2128), 20170350.  **The Limitations of AI in General Practice**  Emanuel, E. J., Wachter, R. M., & Coates, V. (2019). Artificial intelligence in health care: Will the value match the hype? JAMA, 321(23), 2281-2282.  Obermeyer, Z., Powers, B., Vogeli, C., & Mullainathan, S. (2019). Dissecting racial bias in an algorithm used to manage the health of populations. Science, 366(6464), 447-453.  Rajkomar, A., Oren, E., Chen, K., Dai, A. M., Hajaj, N., Hardt, M., Liu, P. J., Liu, X., Marcus, J., Sun, M., Sundberg, P., Yee, H., Zhang, K., & Esteva, A. (2018). Scalable and accurate deep learning with electronic health records. NPJ Digital Medicine, 1(1), 18.  **Ethical Considerations**  Caliskan-Islam, A., Bryson, J. J., & Narayanan, A. (2018). Semantics derived automatically from language corpora contain human-like biases. Science, 356(6334), 183-186.  **Conclusion**  Topol, E. (2019). High-performance medicine: the convergence of human and artificial intelligence. Nature Medicine, 25(1), 44-56. | **Applications of AI in General Practice**  Summerton N. & Cansdale M. (2019). Artificial intelligence and diagnosis in general practice. British Journal of General Practice, 68(684):324-325).  Sørensen, N.L., Bemman, B., Jensen, M.B. *et al.* Machine learning in general practice: scoping review of administrative task support and automation. *BMC Prim. Care* **24**, 14 (2023). <https://doi.org/10.1186/s12875-023-01969-y>  **Applications of AI in General Practice**  <https://bjgp.org/content/69/684/324>  <https://bmcprimcare.biomedcentral.com/articles/10.1186/s12875-023-01969-y>  <https://www.researchgate.net/publication/342052268_The_Future_of_Healthcare_Computerisation_Automation_and_General_Practice_Services>  **The Limitations of AI in General Practice**  <https://www.frontiersin.org/articles/10.3389/fsurg.2022.862322/full>  <https://bjgp.org/content/69/684/324> |
| *Peer reviewed article & low accuracy* | |
|  |  |
| *Not peer reviewed article & high accuracy* | |
|  | **Introduction:**  <https://www.ibm.com/topics/artificial-intelligence-healthcare>  **Applications of AI in General Practice**  <https://www.nature.com/articles/s41569-021-00548-x>  <https://www.who.int/news/item/28-06-2021-who-issues-first-global-report-on-ai-in-health-and-six-guiding-principles-for-its-design-and-use> - Applications for AI in general Practice  **The Limitations of AI in General Practice**  <https://dataconomy.com/2023/01/artificial-intelligence-security-issues/?utm_content=cmp-true>  **Ethical Considerations**  <https://www.forbes.com/sites/arunshastri/2021/05/03/the-art-of-balancing-two-forces-ai-and-human-nature/?sh=371fa87d27fb>  <https://www.forbes.com/sites/forbescommunicationscouncil/2021/08/26/better-together-striking-the-balance-between-artificial-and-human-intelligence/?sh=7c4811be3ab2>  <https://www.who.int/news/item/28-06-2021-who-issues-first-global-report-on-ai-in-health-and-six-guiding-principles-for-its-design-and-use> |
| *Not peer reviewed article & low accuracy* | |
|  |  |
| *Not relevant article* | |
|  |  |
| *Article does not exist* | |
| **The Limitations of AI in General Practice**  Garcia-Rill, E., & Simon, C. (2020). AI in medicine: Privacy and data security. Sleep Science and Practice, 4(1), 1-6.  Kuo, M. H., Sahama, T., & Kushniruk, A. W. (2018). Ethical issues in using healthcare data for artificial intelligence: A systematic review. BMJ Health & Care Informatics, 25(3), e000079.  Shenoy, S., Hasija, S., & Saxena, A. (2019). Data security and privacy in healthcare: Current state of research. International Journal of Computer Applications, 180(46), 1-8.  Beam, A. L., Kohane, I. S., & Berger, B. (2020). Opening the black box of AI in medicine: How transparent are we really? Nature Medicine, 26(6), 841-846.  **Ethical Considerations**  Mikolov, V., Lukaschuk, S., Gonzalez, J. A., & Abujaber, S. (2020). Physician-led artificial intelligence governance: A requirement for responsible innovation. The Lancet Digital Health, 2(10), e471-e473.  Wartella, E., Coyne-Beasley, T., LaVail, K., & St. Clair, R. (2019). Integrating artificial intelligence in health care service: A literature review. Journal of Consumer Health on the Internet, 23(3), 301-312.  Cabitza, F., & Rasoini, R. (2019). Artificial intelligence in healthcare: A hybrid of a hype, high hopes, and hurdles. EClinicalMedicine, 11, 54-56.  **Conclusion**  Wartella, E., Coyne-Beasley, T., LaVail, K., & St. Clair, R. (2019). Integrating artificial intelligence in health care service: A literature review. Journal of Consumer Health on the Internet, 23(3), 301-312. |  |
